# Strong resonance response with ultrahigh quality factor in grating-multilayer systems based on quasi-bound states in the continuum

**DOI:** 10.1038/s41598-022-25945-1

**Published:** 2022-12-12

**Authors:** Yuhang Ruan, Yuke Li, Zhengda Hu, Jicheng Wang, Yixiang Wang

**Affiliations:** 1 School of Science, Jiangsu Provincial Research Center of Light Industrial Optoelectronic Engineering and Technology, Jiangnan University, Wuxi, 214122 China; 2 State Key Laboratory of Millimeter Waves, Southeast University, Nanjing, 210096 China

**Keywords:** Optics and photonics, Physics

## Abstract

Optical bound states in the continuum (BICs) exist in many photonic crystals and periodic structures with a strong resonance and ultrahigh *Q* factor. Such phenomena can be used in the designs of narrowband transmission filters, lasers, and sensors. In this paper, we consider the energy bands of a complex structure consisting of a grating and a multilayer substructure to obtain the position of the BIC in the structure. Hence, the higher *Q* factor can be obtained in the grating-multilayer structure than can be realized in the simple grating geometry. We analyze the wave propagation process in the complex structure and the change in the *Q* value via the use of transmission matrix theory. In addition, the reflectance spectrum is found to exhibit a series of asymmetric line-shapes with different values of the asymmetry parameter, δ, due to the interference between the two channels. One of these channels is the broadband channel, induced by the Fabry–Perot resonance, and the other channel is the narrowband channel, induced by guided mode resonance. Quasi-BICs are seen to transform into BICs as the value of δ is decreased gradually to zero. Therefore, a large Goos–Hänchen shift can be achieved as a result of the high Q factor and quasi-BIC. This work designs a complex structure with ultrahigh *Q* factor and strong resonance properties, which has significant implications for exploring the phenomenon of BICs.

## Introduction

In recent years, localized resonance modes in continuous spectra, i.e., bound states in the continuum (BICs) have attracted considerable research interest. The concept of BICs originated in quantum mechanics and this phenomenon has been recently found in destructive interference in optics. In 1929, von Neumann and Wigner found bound states that can exceed the continuum threshold in Schrödinger’s equation^1,2^. The existence of BICs in photonic crystals and the principle of BICs in practical photonic devices were subsequently reported in experimental work^3,4^. The concept of BICs was later applied to fields within optics, including symmetric protected BICs and Friedrich–Wintgen BICs^5^. Symmetry-protected BICs appear in periodic structures with a highly symmetric Γ-point, whereas Friedrich–Wintgen BICs can cause accidental vanishing of modes^5–7^. As well as these common BICs, some rare types of BICs also exist, including Fabry–Perot BICs^8,9^ and single-resonance BICs^10,11^. BICs have the capacity to confine energy in a particular structure and form a strong optical resonance state with long lifetime. These resonance modes may be located in the continuous spectrum and confined without any radiation; resonance modes are sometimes referred to as embedded eigenvalues or embedded trap patterns^6^. The quality factor (*Q* factor) is an important parameter of BICs; the Q factor indicates the strength of the associated light–matter interactions. BICs with large *Q* factor can be utilized in many applications, including optical modulators^12^, nonlinear optical devices^13^, a wide range of sensors^14,15^, and in optical vortices^16–18^.

Optical BICs often exist in a photonic crystal with certain symmetry characteristic. When the symmetry of structure is broken, the BIC will evolve into a quasi-BIC. Local bound state will couple with an extended state to form a resonance leaky mode^19,20^, which will leak into air and have a high *Q* factor. The appearance of a quasi-BIC is often accompanied by a Fano resonance, which is caused by the interference of two channels^21^. Many investigations show that the reflection phase fluctuates dramatically around the incident angle of the resonance peak, such that an extremely high *Q* factor can lead to a large Goos–Hänchen (GH) shift. This GH shift describes the observation that light incident on the interface between two media exhibits a tiny lateral shift relative to the position predicted in the incident plane; this phenomenon was first observed by Goos and Hänchen in 1947^22^ and explained by Artmann in 1948^23^. The GH shift is proportional to the partial derivative of the reflection phase with respect to the incident angle by the stationary phase method. In the transmission-type resonance, a large GH shift can be obtained due to the resonance of the quasi-BIC^24–32^. The GH shift has been studied in many fields as it incorporates strong light resonance and a high *Q* factor; the areas include lossless or lossy slabs^33,34^, photonic crystals^29,35,36^, and Fano resonances^25^.

Recently, Feng Wu et al. proposed a periodic grating structure with a broken in-plane symmetry and obtained a large GH shift based on ultranarrow resonances^37^. They achieved quasi-BICs in a compound grating waveguide based on the excitation of the resonant guided mode. They also established a relationship between the dispersion relations and the guided mode resonance excitation to suggest the existence of a BIC point. Ye et al. achieved singular points of polarizations in the momentum space considering hexagonal photonic crystals^38^. Wang et al. also achieved a beam shift in a photonic crystal plate with triangular holes^39^. In 2020, Zhu et al. revealed the complex correlation among chiral coupling, optical lateral force, and multipolar effects^40^. However, most previous works discussed BICs in simple structures. In this paper we design a more complex structure that consists of a periodic grating and multilayers, which permits the realization of a relationship between the energy bands and the BICs.

Here we define a key parameter describing the asymmetry of the system, *δ,* which describes the geometry of the structure, i.e., a four-part periodic grating will reduce to two-part periodic grating via a variation in the parameter *δ*. We examine the BIC point by calculating energy bands. In the reflectance spectrum, typical asymmetric Fano line-shapes and ultranarrow resonance can be achieved; this state can be referred to as a quasi-BIC state. Light localization in a quasi-BICs is significant in the realization of high *Q* factor resonances and ultranarrow peaks in the symmetry-protected structure. In addition, adding gratings to the multilayer film is found to realizing a higher *Q* factor than that in the structure composed of only a single grating; this increase can be up to a factor of around about 100. We then discuss the wave propagation in the hybrid structure by using a boundary condition analysis and the transfer matrix method^13,14^. Subsequently, unlike in the case of large GH shifts assisted by the Brewster dip or transmission-type resonances, we obtain a large GH shift that assisted by quasi-BIC and observe that the maximum GH shift can be found at a reflectance peak. The commercial finite element solver COMSOL Multiphysics is used to analyze the eigenmodes and reflection spectrum of the system. We theoretically demonstrate that our structure permits the realization of a higher Q factor than that observed in similar systems and that the energy bands provide a practical tool to find the position of BICs. Our work offers an improved method to design high-performance sensors, surface plasmon resonators, and epsilon-near-zero metamaterial slabs in the future.

## Theoretical calculation based on the transmission matrix analysis

**Figure 1:**
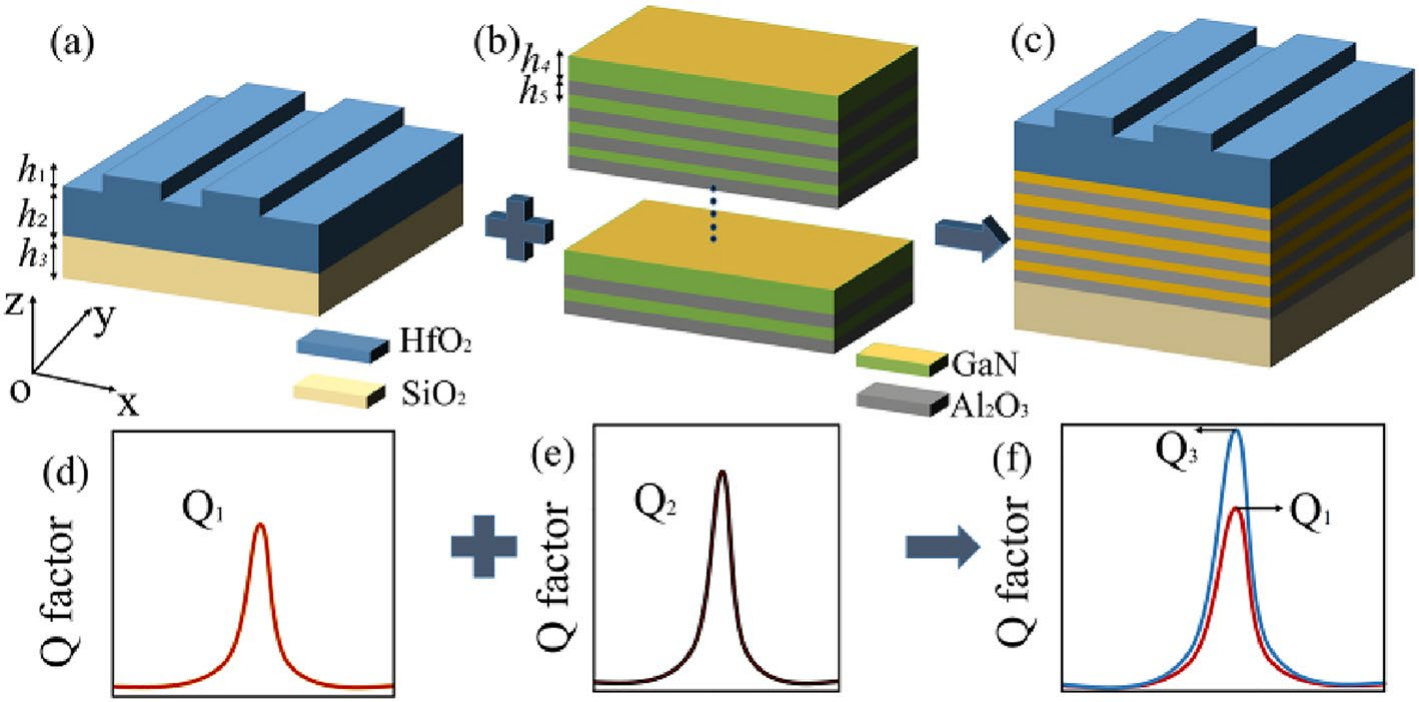
(**a**–**c**) Three-dimensional models of grating, multilayers, and mixed structures. (**d**, **e**) The *Q* value corresponding to the grating and multilayers with *Q*_*1*_ being increased to *Q*_*3*_ via the addition of the multilayer structure.

**Figure 2:**
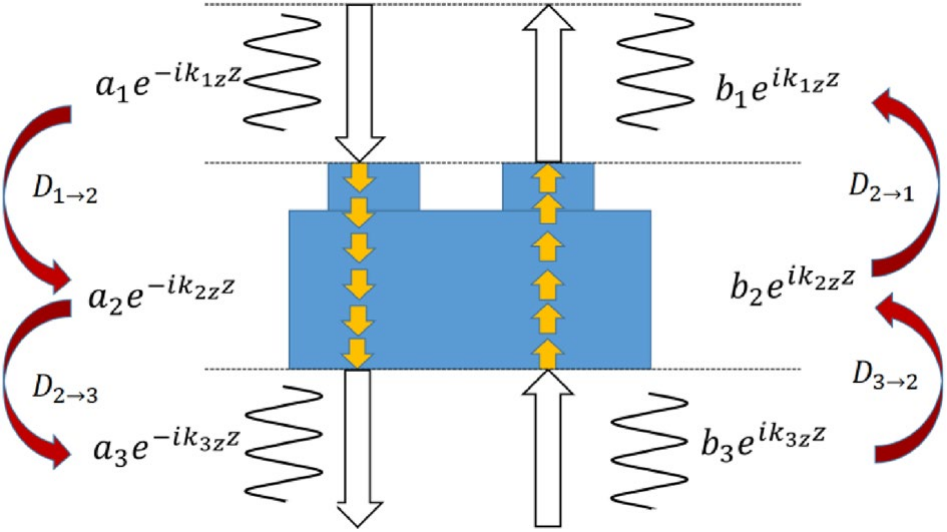
Schematic of the grating and the method used to calculate the wave propagation via the transmission matrix method. The grating is divided into three parts with dielectric constants ε_1_, ε_2_, and ε_3_.

High *([Q]{1,})* factors can be obtained in highly symmetrical gratings, as shown in Fig. 1a and d. Multilayers can also exhibit high *Q* values, as indicated in Fig. 1b and e. When a grating is combined with a multilayer structure to form a new complex structure, a higher *([Q]{1,})* factor can be obtained (see Fig. 1c,f). The schematic diagram of this stacking process is shown in Fig. 1a–c and the associated increase of *Q* factor is shown Fig. 1d and e. In this work, we use the transmission matrix method to theoretically analyze the propagation of light in the structures. We divide the grating into three parts with dielectric constants ε_1_, ε_2_, and ε_3_. Figure 2 shows a schematic of transmission process in the grating structure. The incident wave considered here was y-polarized and propagates parallel to the negative z-axis. This case corresponds to p-polarization, where the propagation direction of the magnetic field is parallel to the y-axis. The incidence magnetic field can be written as follows:

1 $$H_{{1}} y = (a_{{1}} e^{{( - ik_{{1}} zz)}} + b_{{1}} e^{{(ik_{{1}} zz)}} )e^{{(ik_{{1}} xx)}} ,z > 0$$

2 $$H_{{2}} y = (a_{{2}} e^{{( - ik_{{2}} zz)}} + b_{{2}} e^{{(ik_{{2}} zz)}} )e^{{(ik_{{2}} xx)}} ,z < 0$$

3 $$H_{{3}} y = (a_{{3}} e^{{( - ik_{{3}} zz)}} + b_{{3}} e^{{(ik_{{3}} zz)}} )e^{{(ik_{{3}} xx)}} ,z < 0$$

where *a*_*i*_ and *b*_*i*_ (i = 1, 2, 3) are the field coefficients, *k*_*ix*_ and *k*_*iz*_ are the components of wavevectors *k*_*i*_. We can see that *k*_*1x*_ = *k*_*2x*_ = *k*_*3x*_ through Snell’s law. Meanwhile, the boundary condition of the electric and magnetic field at the interface can be determined by using the expressions^14^.

4 $${\varvec{n}} \times \left( {{\varvec{E}}_{2} - {\varvec{E}}_{1} } \right)|_{z = 0} = 0$$

5 $${\varvec{n}} \times \left( {{\varvec{H}}_{2} - {\varvec{H}}_{1} } \right)|_{z = 0} = 0$$

where ***n*** is the unit normal at the interface. Using these boundary conditions we can see that at the interface at *z* = 0 the following expressions hold:

6 $$\frac{{k_{1z} }}{{\varepsilon_{1} }}\left( {a_{1} - b_{1} } \right) - \frac{{k_{2z} }}{{\varepsilon_{2} }}\left( {a_{2} - b_{2} } \right) = 0$$

7 $$\left( {a_{1} + b_{1} } \right) - \left( {a_{2} + b_{2} } \right) = 0$$

where *ε*_*i*_ (*i* = 1, 2, 3) are the dielectric constants. The relationship between the coefficients *a*_1_ and *b*_1_ and *a*_2_ and *b*_2_ can be established by the transmission matrix *D*_1→2_, which is obtained by combining Eqs. (6)–(7). The transmission matrix *D*_1→2_ is calculated to be,

8 $$D_{1 \to 2} = \frac{1}{2}\left[ {\begin{array}{*{20}c} {1 + \eta + \xi } & {1 - \eta - \xi } \\ {1 - \eta + \xi } & {1 + \eta - \xi } \\ \end{array} } \right]$$

where the parameters and are given by,

9 $$\eta = \frac{{\varepsilon_{1} k_{2z} }}{{\varepsilon_{2} k_{1z} }},\;\;\;\xi = 0$$

We then take the thickness of grating into consideration using the propagation matrix, where *Δz* = *h*_1_ + *h*_2_. The transmission process of waveguide layer also needs to be calculated for this structure.

10 $$p = \left[ {\begin{array}{*{20}c} {e^{{ik_{z} \Delta z}} } & 0 \\ 0 & {e^{{ - ik_{z} \Delta z}} } \\ \end{array} } \right]$$

For a more complicated structure, such as the structure shown in Fig. 1c, a new transmission matrix, *D*, must be considered; this transmission matrix is obtained by taking the product of multiple matrices:

11 $$D = D_{1 \to 2} pD_{2 \to 3}$$

**Figure 3:**
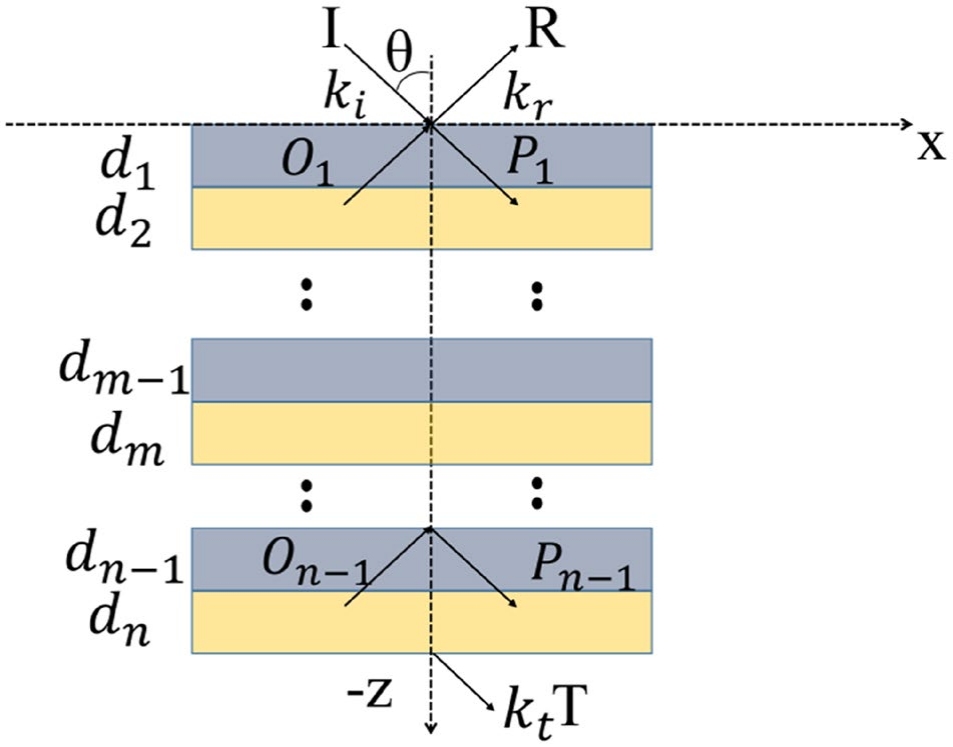
Schematic of the multilayers showing the propagation of light in the various single layers. A high value of transmission, *T*, is the primary aim for this structure.

In this work, we only consider the reflection coefficient, *r*, which is calculated using certain elements of the matrix *D*. We use the expressions,

12 $$r = \frac{{D_{21} }}{{D_{11} }},\;\;\;R = \left| {r^{2} } \right|$$

where *D*_*ij*_ are the elements of *D* and the reflectance is denoted *R*. When the incident wave passes through the grating, we consider a transmission parameter, *T*. For the interface between the two different media, we analyze the propagation of the wave using the transmission matrix. The expressions that describe the electric field different regions of the model can be derived using the following expressions. Here, we list the expressions for the normalized electric field at the input and output regions of the multilayers.

13 $$E_{0} = \left[ {exp\left( {jk_{I, - z} z} \right) + Rexp\left( { - jk_{I, - z} z} \right)} \right]exp\left( { - jk_{x} x} \right),Z \ge 0$$

$$E_{m} = \left\{ {P_{m} exp\left[ { - k_{0} \gamma_{m} \left( { - z - D_{m - 1} } \right)} \right] + Q_{m} exp\left[ {k_{0} \gamma_{m} \left( { - z - D_{m} } \right)} \right]} \right\}*$$

14 $$exp\left( { - jk_{x} x} \right),D_{m} \ge - Z \ge D_{m - 1}$$

15 $$E_{t} = Texp\left\{ { - j\left[ {k_{x} x + k_{II, - z} \left( { - z - D_{n} } \right)} \right]} \right\}, - Z \ge D_{n}$$

16 $$k_{x} = k_{0} n_{I} sin\left( \theta \right),\;\;\;k_{I,z} = k_{0} n_{I} cos\left( \theta \right),$$

17 $$k_{II,z} = k_{0} \left[ {n_{II}^{2} - n_{I}^{2} sin^{2} \left( \theta \right)} \right]^{1/2} ,\theta = 0$$

18 $$\gamma_{m} = j\left[ {n_{m}^{2} - n_{I}^{2} sin^{2} \left( \theta \right)} \right]^{1/2} ,m = 1,2 \ldots ,n,D_{m} = \mathop \sum \limits_{x = 1}^{m} d_{x}$$

where is the incident angle of the wave, *O* and *P* are the field amplitudes in the various single layers, *k*_*0*_ = *2π/λ*_*0*_ is the wave vector, and the refractive indices in the input and output regions are defined as *n*_*I*_, *n*_*II*_. The structure of these regions is shown in Fig. 3.

In the first layer at the boundary *z* = 0, we have,

19 $$1 + R = P_{1} + Oexp\left( { - k_{0} \gamma_{1} d_{1} } \right)$$

20 $$j\left( {k_{I,z} /k_{0} } \right)\left( {1 - R} \right) = \gamma_{1} \left[ {P_{1} - O_{1} exp\left( { - k_{0} \gamma_{1} d_{1} } \right)} \right]$$

At the interface between the (*m*-1)-th and *m*-th layer, the relevant expressions can be written as follows,

21 $$P_{m - 1} exp\left( { - k_{0} \gamma_{m - 1} d_{m - 1} } \right) + O_{m - 1} = P_{m} + O_{m} exp\left( { - k_{0} \gamma_{m} d_{m} } \right)$$

22 $$\gamma_{m - 1} \left[ {P_{m - 1} exp\left( { - k_{0} \gamma_{m - 1} d_{m - 1} } \right) - O_{m - 1} } \right] = \gamma_{m} \left[ {P_{m} - O_{m} exp\left( { - k_{0} \gamma_{m} d_{m} } \right)} \right]$$

At the output of the structure, the relevant equations are,

23 $$P_{n} exp\left( { - k_{0} \gamma_{n} d_{n} } \right) + O_{n} = T$$

24 $$\gamma_{n} \left[ {P_{n} exp\left( { - k_{0} \gamma_{n} d_{n} } \right) - O_{n} } \right] = j\left( {k_{II,z} /k_{0} } \right)T$$

Solutions for *R* and *T* can be obtained using the entire system of equations. The transmission matrix approach shown in Eqs. (23) and (24) is used to calculate the field amplitudes *P*_*n*_ and *O*_*n*_ for the transmittance coefficient *T*.

25 $$\left[ {\begin{array}{*{20}c} {P_{n} } \\ {O_{n} } \\ \end{array} } \right] = \left[ {\begin{array}{*{20}c} {exp\left( { - k_{0} \gamma_{n} d_{n} } \right)} & 1 \\ {\gamma_{n} exp\left( { - k_{0} \gamma_{n} d_{n} } \right)} & { - \gamma_{n} } \\ \end{array} } \right]^{ - 1} \left[ {\begin{array}{*{20}c} 1 \\ {j\left( {k_{II,z} /k_{0} } \right)} \\ \end{array} } \right]T$$

Substituting these expressions into Eqs. (21) and (22), we obtain the field amplitudes *P*_*n-1*_ and *O*_*n-1*_. Repeating this process for all the layers within the structure, we obtain,

$$\left[ {\begin{array}{*{20}c} 1 \\ {j\left( {k_{I,z} /k_{0} } \right)} \\ \end{array} } \right] + \left[ {\begin{array}{*{20}c} 1 \\ { - j\left( {k_{I,z} /k_{0} } \right)} \\ \end{array} } \right]R = \mathop \prod \limits_{m = 1}^{n} \left[ {\begin{array}{*{20}c} 1 & {exp\left( { - k_{0} \gamma_{m} d_{m} } \right)} \\ {\gamma_{m} } & { - \gamma_{m} exp\left( { - k_{0} \gamma_{m} d_{m} } \right)} \\ \end{array} } \right]*$$

26 $$\left[ {\begin{array}{*{20}c} {exp\left( { - k_{0} \gamma_{m} d_{m} } \right)} & 1 \\ {\gamma_{m} exp\left( { - k_{0} \gamma_{m} d_{m} } \right)} & { - \gamma_{m} } \\ \end{array} } \right]^{ - 1} \left[ {\begin{array}{*{20}c} 1 \\ {j\left( {k_{II,z} /k_{0} } \right)} \\ \end{array} } \right]T$$

When *m* = *n* we have the special case of,

27 $$\left[ {\begin{array}{*{20}c} {exp\left( { - k_{0} \gamma_{n} d_{n} } \right)} & 1 \\ {\gamma_{n} exp\left( { - k_{0} \gamma_{n} d_{n} } \right)} & { - \gamma_{n} } \\ \end{array} } \right]^{ - 1} = \left[ {\begin{array}{*{20}c} {exp\left( { - k_{0} \gamma_{n} d_{n} } \right)} & 0 \\ 0 & 1 \\ \end{array} } \right]^{ - 1} \left[ {\begin{array}{*{20}c} 1 & 1 \\ {\gamma_{n} } & { - \gamma_{n} } \\ \end{array} } \right]^{ - 1}$$

which is the product of two matrices being equivalent to the original inverse matrix. Where *f*_*L*+*1*_ = 1 and *g*_*L*+*1*_ = *jk*_*II,z*_*/k*_*0*._ Using the expression *T* = *exp(− k*_*0*_*γ*_*n*_*d*_*n*_*)T*_*n*_, Eq. (25) can be simplified.

28 $$\left[ {\begin{array}{*{20}c} {a_{n} } \\ {b_{n} } \\ \end{array} } \right] = \left[ {\begin{array}{*{20}c} 1 & 1 \\ {\gamma_{n} } & { - \gamma_{n} } \\ \end{array} } \right]^{ - 1} \left[ {\begin{array}{*{20}c} {f_{n + 1} } \\ {g_{n + 1} } \\ \end{array} } \right]$$

29 $$\begin{aligned} \left[ {\begin{array}{*{20}c} {f_{n} } \\ {g_{n} } \\ \end{array} } \right]T_{n} & = \left[ {\begin{array}{*{20}c} 1 & {exp\left( { - k_{0} \gamma_{n} d_{n} } \right)} \\ {\gamma_{n} } & { - \gamma_{n} exp\left( { - k_{0} \gamma_{n} d_{n} } \right)} \\ \end{array} } \right]\left[ {\begin{array}{*{20}c} {a_{n} } \\ {b_{n} exp\left( { - k_{0} \gamma_{n} d_{n} } \right)} \\ \end{array} } \right]T_{n} \\ & = \left[ {\begin{array}{*{20}c} {a_{n} + b_{n} exp\left( { - 2k_{0} \gamma_{n} d_{n} } \right)} \\ {\gamma_{L} \left[ {a_{n} - b_{n} exp\left( { - 2k_{0} \gamma_{n} d_{n} } \right)} \right]} \\ \end{array} } \right]T_{n} \\ \end{aligned}$$

It is then possible to obtain the relationship between reflectivity and transmissivity, as well as the final expression for the transmissivity:

30 $$\left[ {\begin{array}{*{20}c} 1 \\ {j\left( {k_{I,z} /k_{0} } \right)} \\ \end{array} } \right] + \left[ {\begin{array}{*{20}c} 1 \\ { - j\left( {k_{I,z} /k_{0} } \right)} \\ \end{array} } \right]R = \left[ {\begin{array}{*{20}c} {f_{1} } \\ {g_{1} } \\ \end{array} } \right]T_{1}$$

31 $$T = exp\left( { - k_{0} \gamma_{n} d_{n} } \right) \cdots exp\left( { - k_{0} \gamma_{m} d_{m} } \right) \cdots exp\left( { - k_{0} \gamma_{1} d_{1} } \right)T_{1}$$

When the light passes through the grating and the multilayer structure, the transmissivity *T* will reduce due to the small value of *exp(− k*_*0*_*γ*_*m*_*d*_*m*_*)*. For this complex structure, we have *R* = *1 – A − T*, where the absorbance, *A*, is equal to zero. With the analysis of the transfer matrix, we can realize transmission and reflection of the whole structure and it can produce a ultra-high reflection due to a low transmission, the asymmetric Fano reflection peak corresponds to the high *Q* factor of the structure. In the following, we confirm this phenomenon via simulations undertaken by using COMSOL Multiphysics.

## Simulations results

**Figure 4:**
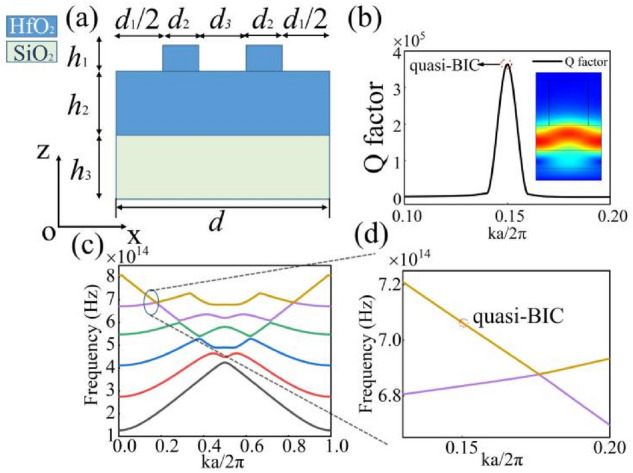
(**a**) A schematic of the grating in the *x-o-z*-plane. (**b**) A high *Q* factor of the optical bound states in the continuum (BIC) can be seen. The inset shows the electric field predominantly existing within the waveguide layer. (**c**) The energy bands of the grating are calculated via a mode analysis, where an optical singularity close to the point of the BIC can be seen. (**d**) The details of the BIC can be seen in the zoomed in region of the energy band diagram.

The grating structure consists of a periodic grating layer and a waveguide layer, as shown in Fig. 4a. The first layer is a grating with a period of *d* = 400 nm and a height of *h*_1_ = 290 nm. The waveguide layer is situated below the grating with a height of *h*_2_ = 160 nm. In this work we use high-index HfO_2_, which has a refractive index, n_HfO2_, of 1.975. The width of grating region of the structure has a constant value of *d*_2_ = 0.2*d*; we use two different values to take into account the air gap in the unit cell: *d*_3_ = *d*_*0*_ + *Δd* and *d*_1_ = *d*_*0*_* − Δd* where *d*_0_ is equal to 0.3*d*. We define an asymmetric geometric parameter, *δ* = *Δd/d*_0_ [− 1, 1], to consider the difference between the second and fourth region in the grating layer. The period of the structure is oriented parallel to the *x*-axis, and the *x-o-z*-plane is chosen to be the incident plane. We can obtain the in-plane symmetry of the grating in *x-o-y*-plane; the *z*-axis is normal to the grating. We set up the projection energy band surface along the x-direction, the periodicity conditions of both sides are Floquet periodicity. Projected energy band scan according to the size of the first Brillouin zone 2*π/d*.

Here, we only consider the parameter *δ* = 0 in the structure without the breaking symmetry in *x-o-y*-plane; in this case *d*_3_ is equal to *d*_1_ and *Δd* = 0. Figure 4b shows an ultrahigh *Q* factor and an electrical field distribution main concentrating in the waveguide layer. The electrical field distribution can be coupled to the extended modes in the continuum very well, which indicates that it is possible to realize the capacity for the grating to carry optical bound states in continuous media. It is noted that the periodic geometry will lead to BIC at the Γ-point of the photonic band structures. The energy bands of the structure are shown in Fig. 4c, which have been obtained by using a mode analysis. The region close to the point of the BIC is shown in Fig. 4d. It is seen that the BIC is not located at the crossing point of the energy bands.

**Figure 5:**
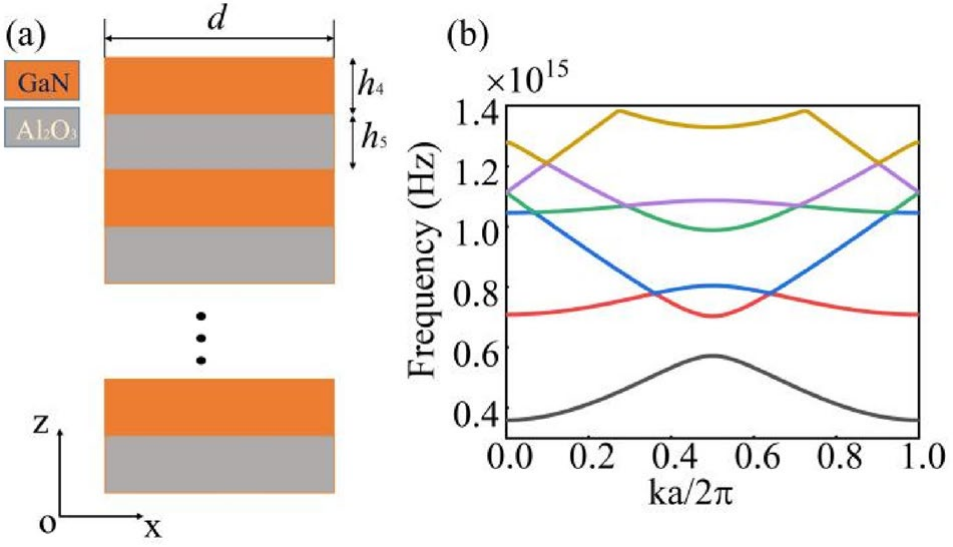
(**a**) Schematic of the multilayers considered here, we apply Floquet periodicity conditions at the both sides of structure. (**b**) The two-dimensional structure of energy bands based on scanning of the Brillouin zone with the proposed multilayers.

We also obtain the analytical results for the multilayer films. The multilayers consist of two different media. GaN, which has a high refractive index of 2.45, making up the top region of the structure, and Al_2_O_3_ is used as the second material in the structure, which has a refractive index of 1.77. For the former, we choose a height of *h*_4_ = 57.25 nm, whereas the latter has a height of *h*_5_ = 82.47 nm. In this work, we define a new combination *H* = *h*_4_ + *h*_5_ as a periodic unit cell of multilayers and decide the number of periods *n* = 2, the number of multilayers does not have much influence for BIC on the structure. In the Fig. 5a, Floquet periodicity conditions are used at both sides of structure, and the size of first Brillouin zone is found to be *2π/d*, as established by the mode analysis. The energy bands of the multilayers are shown in Fig. 5b.

**Figure 6:**
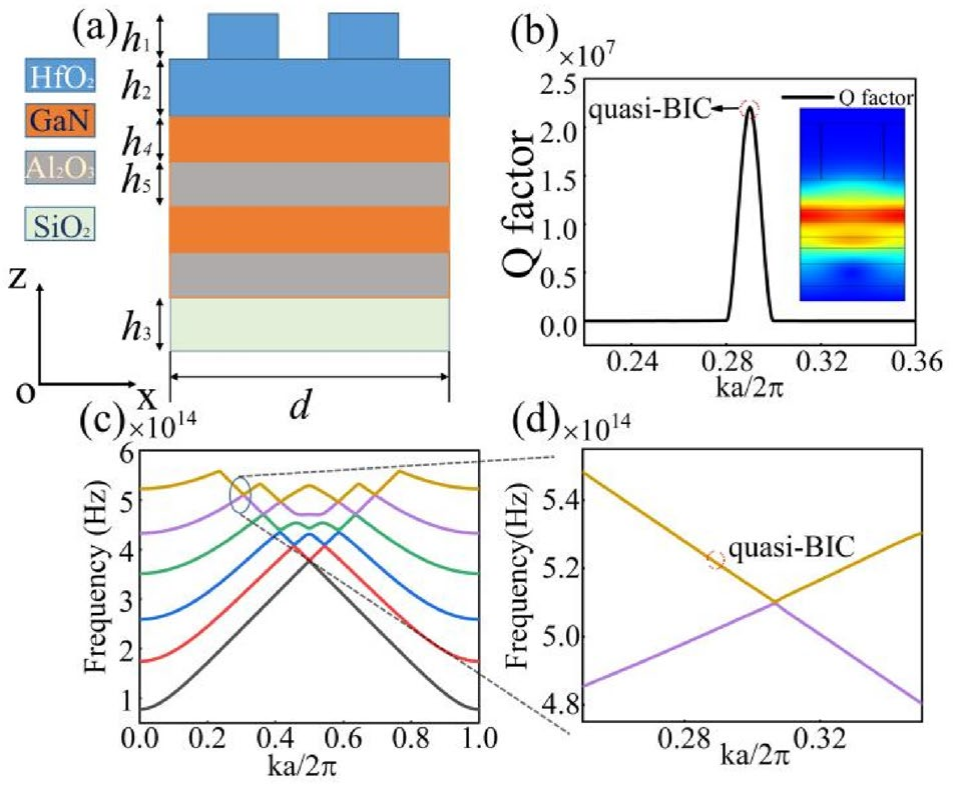
(**a**) Schematic showing the complex structure considered here that is made up of grating and multilayers substructures. (**b**) *Q* factor of this structure was found to be over 107 and the normalized electric field is shown in the inset. (**c**) The energy bands of the hybrid structure with the point of the quasi-BIC indicated near the first crossing point. (**d**) The point of the BIC is located at a *ka/2π* value between 0.28 and 0.32.

In order to obtain a higher *Q* factor, we stack two different photonic crystals to construct a complex structure, as shown in Fig. 6a. It is found that a higher *Q* factor can be achieved comparing to the pervious structure, as shown in Fig. 6b; we note that different energy bands can be observed. The energy bands of this structure, as shown in Fig. 6c, have many features in common with the energy band structure of the grating structure considered above. The location of the BIC is closer to the middle point than was found in the case for the grating structure alone. The main function of the energy band is to find the location of BIC and match the position of reflection peak. We can calculate resonant wavelength from energy bands *λ* = *c/f*, with calculation, wavelength *λ* is approximately equals to 660 nm. Details of the quasi-BIC can be observed in Fig. 6d with the energy bands. Meanwhile, it is found that the electric field is enhanced and localized in the grating. For better understand the transformation of the BIC and the quasi-BIC, we also calculate the reflectance spectra of the structure. The reflection phenomenon and asymmetric Fano line-shapes can be got where it states that the cause of these line-shapes is based on channel interference theory. Fano line-shapes will be dependent on the value of *δ*, i.e., with an asymmetric geometric parameter *δ* varying from a nonzero value to zero then the Fano line-shapes will disappear.

**Figure 7:**
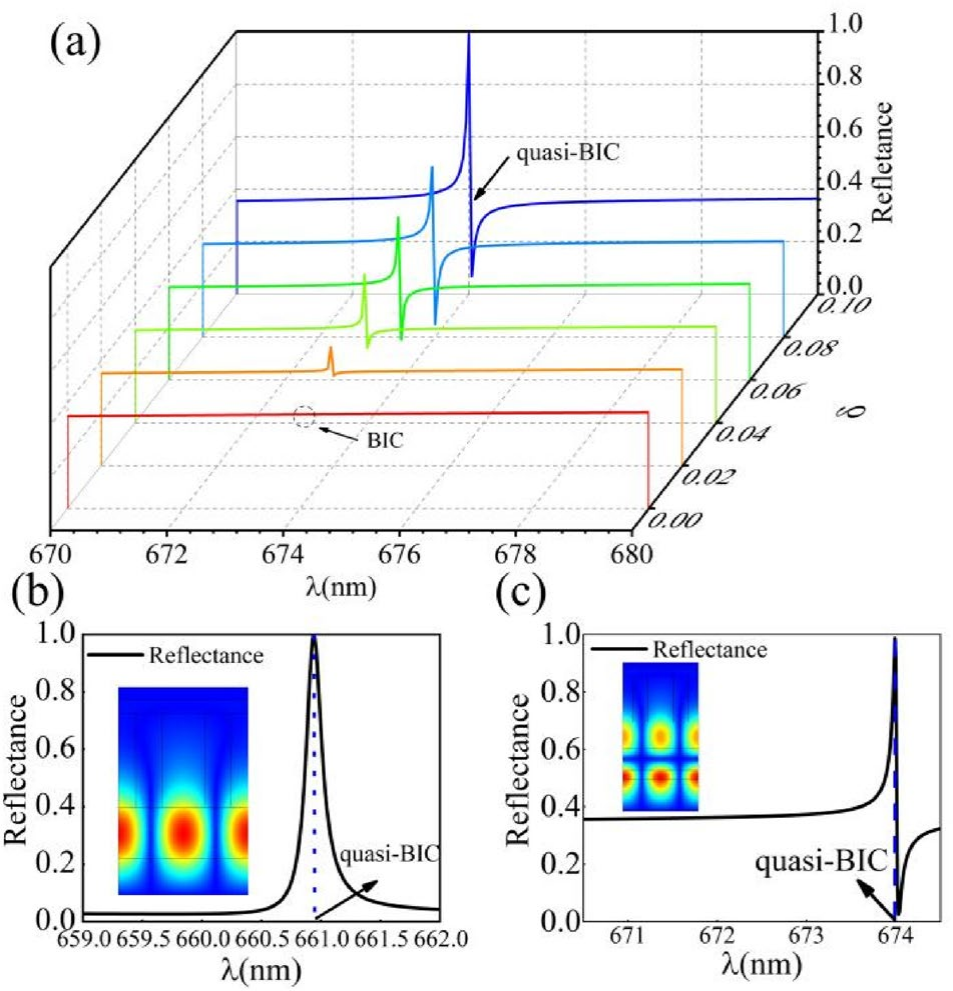
(**a**) The value of reflectance for various values of *δ* and wavelength. The value of *δ* is changed from 0 to 0.1 which can explain the appearance of the quasi-BIC. When the *δ* decreases to 0, the resonance peak disappears gradually, which signifies the appearance of the BIC. (**b**) The reflectance spectra of the grating for wavelengths from 659 to 662 nm. (**c**) A sharp resonance peak appears at the quasi-BIC is shown in the reflectance spectra of the mixed structure; the reflectance is plotted for values of wavelength from 670 to 675 nm.

We obtain the Q value by calculating the reflectance spectra^41^, using the expression *([Q]{1,})* = *λ*_*peak*_*/Δλ* where *Δλ* =*|λ*_*dip*_* − λ*_*peak*_*|*. A series of narrow reflection resonances are shown in Fig. 7a, where the resonance phenomena can be attributed to interactions between continuous state and discrete state in reflectance spectra. Quasi-BIC indicates that the ultranarrow resonance width tends to zero as the asymmetrical parameter varies continuously in reflectance spectra. When the value of *δ* is changed from 0 to 0.1, a small change in the parameter value leads to the disappearance of modes. Prominent resonant features are clearly visible in Fig. 7a that corresponds to the existence of trapped electromagnetic modes. Interference between discrete resonant states of different channels explain the appearance of asymmetric Fano line-shapes, which are caused by the broadband channel and narrowband channel. In this work, we have utilized the transmission matrix method to establish the reflection characteristics of a complex structure. For *δ* = 0.1, a perfect Fano line is obtained, which has an ultranarrow resonance width and ultrahigh reflectance. If *δ* is lower than 0.1, the line-shape becomes smaller and the resonance gradually disappear with *δ* decrease. In addition, the resonance width becomes lager when the value of *δ* is large than 0.1. The quasi-BIC can be illustrated by calculating the energy bands and spectrum of the resonator for a grating structure with an asymmetric parameter of *δ* = 0.1.

An ultrahigh resonance peak can be seen at a wavelength of 660.9 nm in Fig. 7b, which corresponds to a structure of grating only; in that figure, the electric field distributions at the reflectance peak are plotted in the inset. The grating is a four-part periodic structure with a quasi-BIC when *δ* = 0.1. Here, it is found that the electric field can converge to the waveguide layer with a quasi-BIC. The electric field concentrates in the waveguide layer at a wavelength of 660.9 nm. In this case, the mode represents leak and loss then the radiation will propagate into free-space, which leads to a finite lifetime rather than infinite lifetime of the BIC.

For a more complicated structure, the point of the resonance peak is seen to shift toward longer wavelengths, as shown in Fig. 7c. In this case a resonance peak locates at a wavelength of *λ* = 674.0 nm, where a sharp asymmetric Fano line-shape can be observed along with an ultranarrow linewidth. The reflection peak is obtained closing to complete reflection, and it is in good agreement with the wavelength calculated by energy band (660 nm). At the same time, the electric field becomes more localized in the waveguide layer, which also indicates that the resonance becomes stronger for these parameter values. When the value of *δ* gradually decreases from 0.1 to 0, the resonance width reduces dramatically. When the value of *δ* equals to 0, the width of the resonance vanishes and a dark mode appears with an infinite *Q* factor. This state corresponds to BIC for a smooth reflection curve. The *Q* value can be calculated by using the formula *([Q]{1,})* = *λ*_*peak*_*/Δλ* and the multiplier of *Q* value is only around 30.59, which is different from that for structures in which true BICs exist.

**Figure 8:**
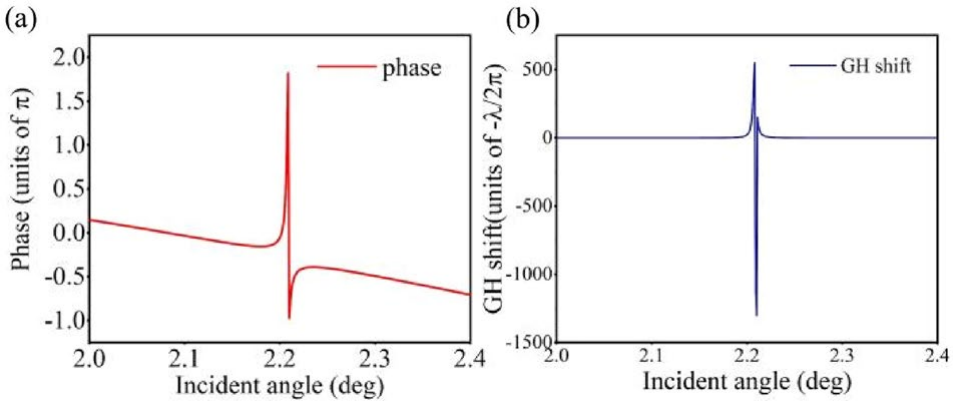
(**a**) TE reflection phase angular spectra with *λ* = 674.0 nm. (**b**) GH shift angular spectra with the asymmetric geometric parameter set to *δ* = 0.1.

The complex structure consists of grating and multilayers has an ultrahigh *Q* factor, which can achieve a large GH shift. Next, we utilize the quasi-BIC to achieve a large GH shift based on a phase change. The asymmetry geometric parameter *δ* determines the value of the GH shift, i.e., resonance will become stronger and result in a larger GH shift for smaller values of the asymmetric parameter. Here the asymmetric geometric parameter is set to *δ* = 0.1, phase changes with incident angle increase under *λ* = 674.0 nm, which can be seen in Fig. 8a. The resonance width in the angular spectrum is ultra-narrow, due to the strong resonance of the quasi-BIC. Here, for the stationary phase method, the lateral GH shift for the reflected and transmitted beams can be calculate by using the expression^29,34^,

32 $$S_{{{\text{GH}}}} = - \frac{\lambda }{{{2}\pi }}\frac{{\partial_{{\phi_{{\text{r}}} }} }}{{\partial_{\theta } }}$$

where *φ*_*r*_ is the reflection phase and the GH shift is proportional to the partial derivative of the reflection phase. The GH shift angular spectra is shown in Fig. 8b. When the incident angle is far from the resonance angle of *θ* = 2.2°, the reflected phase remains smooth. However, the GH shift shows significantly different behavior around the resonance angle of *θ* = 2.2°. In contrast with traditional GH shift enhanced by transmission-type resonances, the maximum GH shift assisted by the quasi-BIC locates at a reflectance peak and can be detected and utilized more easily.

## Conclusions

In conclusion, we have designed a complex structure with a high *Q* factor. We employ the finite element method to find the point of the BIC in the energy bands. We use the transmission matrix method to analyze the propagation of light and the change in *Q* factor compare to other structures, which we evaluated quantitively. The asymmetric parameter *δ* controls the geometry of structure, i.e., a four-part periodic grating reduces to two-part periodic grating by varying the value of *δ*. When the grating retains its symmetry with *δ* = 0, asymmetric Fano resonance peak that corresponds to the appearance of BIC are not observed. With an increase of *δ*, a typical asymmetric Fano line-shape is observed and strong resonance achieved by the quasi-BIC. Adding a periodic multilayers structure to the grating geometry result in a higher *Q* factor and a narrower Fano line-shape. Assisted by ultrahigh the *Q* value and narrow resonance line width, a saltatory phase can be obtained and the value of the GH shift is greatly increased. The maximal GH shift locates at the reflectance peak that is different from induced by transmission-type resonances or the Brewster dip. Our results provide a new method to realize BICs, which may provide the design of high-performance sensors, wavelength division multiplexers, and optical switches.

## Data Availability

The data and material that support the findings of this study are available from the corresponding author upon reasonable request.

## Code availability

The code that support the findings of this study are available from the corresponding author upon reasonable request.
